# Worries and information seeking during pregnancy: a cross-sectional study among 1402 expectant Norwegian women active on social media platforms

**DOI:** 10.1080/02813432.2025.2461036

**Published:** 2025-02-15

**Authors:** Mari Hegnes Hansen, Hanna Sandbakken Mørkved, Bjarne Austad, Gunnhild Åberge Vie, Linn Okkenhaug Getz, Bente Prytz Mjølstad

**Affiliations:** Department of Public Health and Nursing, Faculty of Medicine and Health Sciences, General Practice Research Unit, Norwegian University of Science and Technology, NTNU, Trondheim, Norway

**Keywords:** Pregnancy care, worries, information seeking, social media, survey

## Abstract

**Background:**

Pregnant women often navigate extensive information from healthcare professionals, social networks and online sources, which can increase anxiety. Understanding their concerns and preferred information sources is crucial for effective antenatal care.

**Objective:**

To explore worries and information-seeking behaviour among pregnant women in Norway using social media.

**Methods:**

An anonymous, web-based survey was conducted among 1402 pregnant women in Norway from January to March 2022, distributed via Facebook and Instagram. The survey covered 11 pregnancy worries, eight postpartum worries, information sources and thoughts regarding childbirth.

**Results:**

Nearly, all participants had concerns, mainly about foetal anomalies (99%, *n* = 1381), miscarriage (95%, *n* = 1332) and childbirth (85%, *n* = 1195). Postpartum worries included physical changes (90%, *n* = 1266), breastfeeding (85%, *n* = 1187) and financial situation (74%, *n* = 1030). Major worries were more common among first-time mothers and women with financial insecurity. Most women sought information from quality-assured public health websites (74%, *n* = 1042) and healthcare personnel (56%, *n* = 775), with only 2% (*n* = 32) turning to influencers or bloggers.

**Conclusions:**

Pregnancy and postpartum worries are widespread among pregnant women using social media, especially among first-time mothers and those with financial insecurity. Most pregnant women prefer quality-assured websites and healthcare personnel for information. Antenatal care could benefit from offering more tailored information and follow-up, particularly for first-time mothers and financially insecure women.

## Background

Pregnancy is a period in life when concerns easily arise, related to both the health of the pregnant woman and the foetus. While important harmful factors such as smoking and alcohol consumption are well known and in principle easy to avoid, more recent research on risk factors that may affect the foetus, has led to an increasing number of precautions advised for pregnant women [1,2].

The infant mortality rate in Norway is among the lowest in the world (1.6 deaths per 1000 live births) [3] and most pregnancies proceed without significant complications (93% and 69%, respectively, of multi- and primipara) [4]. Still, pregnancy-related worries are common and there is a tendency to over-monitor pregnant women in Norway [5]. Concerns, worries and anxiety can be viewed as points on a continuum, with anxiety disorders that significantly impact daily life positioned at the higher end of the scale [6]. Previous research has shown that widespread worries related to fear of miscarriage, the baby’s health and giving birth have been relatively stable over time, across different European countries [6–8]. Unplanned pregnancies, previous miscarriages and first-time pregnancies seem to influence the levels of concern [9].

Although pregnant Norwegian women appear to lead healthier lives than before in terms of diet, smoking and alcohol consumption [10,11], increased risk awareness could also heighten worries. Approximately, 55,000 Norwegian women give birth each year and the average age of mothers has risen to 31.4 years in 2020 [4]. This increase in maternal age could potentially influence lifestyle choices and both actual and perceived risks. Socioeconomic status and degree of social support are also known factors that can affect the pregnancy and women’s experiences [12,13].

The Norwegian welfare system offers substantial financial benefits to pregnant women, and antenatal care is well-organised and widely regarded as high-quality [1]. The characteristics of the current antenatal care programme are described in Textbox 1. Norwegian guidelines recommend structured discussions about lifestyle [1]. Additionally, quality-assured information about pregnancy and antenatal care is easily accessible on public health information websites.

Textbox 1. Characteristics of current Norwegian antenatal care as described in the National guideline [1].

- All pregnant women living in Norway are entitled to free antenatal care.
- The routine programme is identical for all women regardless of previous childbirth and includes nine controls.
- The first visit is recommended in weeks 6–8 (‘as early as possible’) in order to address lifestyle issues.
- The programme includes ultrasound scans in pregnancy week 12 (gradually implemented since 2021) and weeks 17–19 (established in the early 1990s).
- Routine antenatal care is mainly provided by midwives and/or regular general practitioners (GPs).
- The women can choose whether they prefer antenatal consultations with their GP, a midwife or both. Due to the regular GP scheme, which ensures all Norwegian citizens the right to a regular GP, most GPs are involved in antenatal care.
- High-risk or complicated pregnancies are referred to specialised healthcare, but may still be partly followed up in primary care.

The use of internet and social media among pregnant women is widespread, and pregnant women often report considering the information to be reliable and useful [14–16]. Primipara, younger women and highly educated women are the most likely to use the internet for information seeking, and topics most frequently searched for are foetal development, pregnancy complications, childbirth and maternal health [17]. Many use the internet instead of talking to their healthcare provider, so the quality of the information is important [15–17]. Expectant women seem to use and rely on social media to a greater extent than more formally quality assured sources [17]. A systematic review found that social media and mobile health apps could be effective in promoting the mother’s health [18]. However, social media are complex and highly dynamic arenas, involving forceful cultural processes and commercial actors [16]. In the digital landscape, several ‘influencers’ blog about their own individual experiences and give advice related to pregnancy and parenthood. A recent systematic review was not able to conclude whether engaging with such influencers was beneficial or harmful for pregnant women [19]. Influencers might share useful information, but also misinformation, provide support and shape norms and perceptions [19]. Another important source of information is advice from family and friends, but the extent to which women seek such information and how they experience its usefulness, is unknown [20].

There is little updated, research-based knowledge about expectant women’s common concerns and worries and how they seek and value different sources of information. The aim of this study was to explore these common worries among pregnant women in Norway who use social media.

## Materials and methods

### Study design and setting

Between January and March 2022, an anonymous, electronic cross-sectional survey was conducted among pregnant women over 18 years old in Norway. Participants were recruited through Facebook and Instagram, which in addition to Snapchat are the most frequently used social media platforms among Norwegian adults [21]. We identified nine ongoing Facebook groups for pregnant women (so-called ‘Due date’ groups) of which six groups with a total of 10,500 members allowed us to distribute the survey. Furthermore, the survey was distributed on the five most popular ‘Pregnancy and childbirth’ – Instagram accounts ranging from 5500 to 44,000 followers. For comparison, only 51,480 women gave birth in Norway in 2022. The study was promoted through a short video and postings about the study. Participants consented by answering the questionnaire, which was in Norwegian, introduced by a formal information letter on the first page. The study followed The Checklist for Reporting Results of Internet E-Surveys (CHERRIES) for online surveys, which includes quality assessments regarding design, ethics, analysis and publication [22].

### The questionnaire

Due to the lack of a suitable pre-existing questionnaire for our study, we developed a new one, incorporating items from validated questionnaires where possible. While several items are similar to the Cambridge Worry Scale [7,8], we included additional lifestyle-related topics often discussed during antenatal check-ups, excluding those less relevant in the Norwegian context. Worries related to personal finances were formulated as the inability to handle unexpected expenses.

Ten pregnant women were invited to pilot the survey to assess the completion time and clarity of the questions. Six women responded. Apart from suggestions of minor linguistic adjustments, the piloting led to the addition of ‘physical changes of the body’ as a new post-birth concern. We used a data collection tool (Nettskjema.no), which is user-friendly and compatible with computers, smartphones and tablets. The questionnaire contained 48 items. Seven of these were open-ended, and the rest multiple-choice. The survey was divided into five parts: background/demographic variables (previous pregnancies/births, trimester), physical and mental health, worries, antenatal care (information and advice), information sources and thoughts regarding childbirth and the parental role. The present study reports findings regarding worries and information seeking.

### Study variables

Participants were asked about 11 specific worries up to the current stage of their pregnancy, as well as eight worries related to the situation after delivery. We chose to use a five-point Likert scale that is easy to understand, interpret and analyse. The five response categories ranged from ‘not at all’, to a ‘very great extent’. In the analysis, we merged ‘great/very great extent’ to indicate major worries and ‘some/small extent’ to indicate minor worries. We further enumerated how many major worries each woman reported (of the 11 specific worries during pregnancy and the eight specific worries related to the situation after delivery).

As it was possible to answer multiple categories of main employment status, we manually categorised some of the respondents into the most likely category (see Additional file A1).

We did not inquire about income, instead we asked whether the women would be able to handle an unforeseen expense of NOK 10,000 (approximately EUR 1000) [23]. We merged ‘no’ and ‘uncertain’ and defined these responses as financial insecurity.

Regarding information sources, we asked where the women generally sought advice and support regarding the pregnancy, and which information sources they accessed if they had specific questions or wanted to learn more about pregnancy-related topics. Response categories ranged from ‘not at all’ to ‘to a very great extent’, and we merged responses to ‘great/very great extent’, ‘some/small extent’ and ‘not at all’.

### Statistical analysis

Data were analysed using SPSS Statistics version 29 (SPSS Inc., Chicago, IL). We present descriptive statistics for the total sample and for subgroups defined by parity, financial security, age and trimester. We calculated mean and proportion for subgroups of samples with 95% CI. Pearson’s Chi-squared test was used to examine associations between major worries and parity, as well as between major worries and financial security. Missing data were excluded from the analysis.

## Results

### Participants’ characteristics

A total of 1402 expectant women participated in the study. Of these, 868 (62%) were recruited from pregnancy groups on Facebook and 534 (38%) from Instagram.

Characteristics of the study population are presented in Table 1. The majority were between 25 and 37 years of age, in a relationship (married, cohabiting or partner) and employed. Half of the respondents were pregnant for the first time (52%, *n* = 722) and in their second trimester (55%, *n* = 776). Over 95% of the respondents (*n* = 1332) reported having a Norwegian background. Most items had less than three missing answers. The lowest number of responses to one single item was 1390 of 1402.

**Table 1.** Demographics and characteristics of the study population (*n* = 1399–1402).

Variables		*n*	%
Age (years)	<25	119	8.5
	25–37	1226	87.4
	>37	57	4.1
Parity	Primipara	722	51.5
	Multipara	680	48.5
Gravida^a^	1	553	39.5
	2–3	723	51.6
	More than 3	124	8.9
Gestational age^a^	First trimester	99	7.1
	Second trimester	776	55.4
	Third trimester	525	37.5
Marital status	Living with spouse/partner	1369	97.6
	Not living with spouse/partner	33	2.4
Education	Primary school	28	2.0
	High school	315	22.5
	College/university – less than 4 years	495	35.3
	College/university − 4 years or more	564	40.2
Employment status	Employed	1210	86.3
	Student/apprentice	123	8.8
	Unemployed (looking for employment)	12	0.9
	Receiving welfare benefits	44	3.1
	Housewife	13	0.9
Ability to handle an unforeseen expense of EUR 1000^a^	Yes	1166	83.3
	No/do not know	234	16.7
Residence^b^	Northern Norway	129	9.2
	Central Norway	230	16.4
	Western Norway	323	23.1
	Eastern Norway	631	45.1
	Southern Norway	87	6.2
Size of municipality^b^	Rural area (less than 5000 inhabitants)	270	19.3
	Town (5000–20,000 inhabitants)	331	23.6
	City (more than 20,000 inhabitants)	800	57.1
Driving distance to hospital^b^	Less than 1 h	1203	85.9
	1–2 h	140	10.0
	More than 2 h	58	4.1
Birthplace	Norway	1336	95.3
	Another country	66	4.7
Cultural background^c^	Norwegian	1332	95.2
	Not Norwegian	15	1.1
	Multicultural	52	3.7
Mother tongue^c^	Norwegian	1341	95.9
	Another Nordic language	24	1.7
	Another European language	20	1.4
	Another non-European language	14	1.0

^a^ Missing values 2.

^b^ Missing value 1.

^c^ Missing values 3.

### Worries in pregnancy

**Figure 1.**
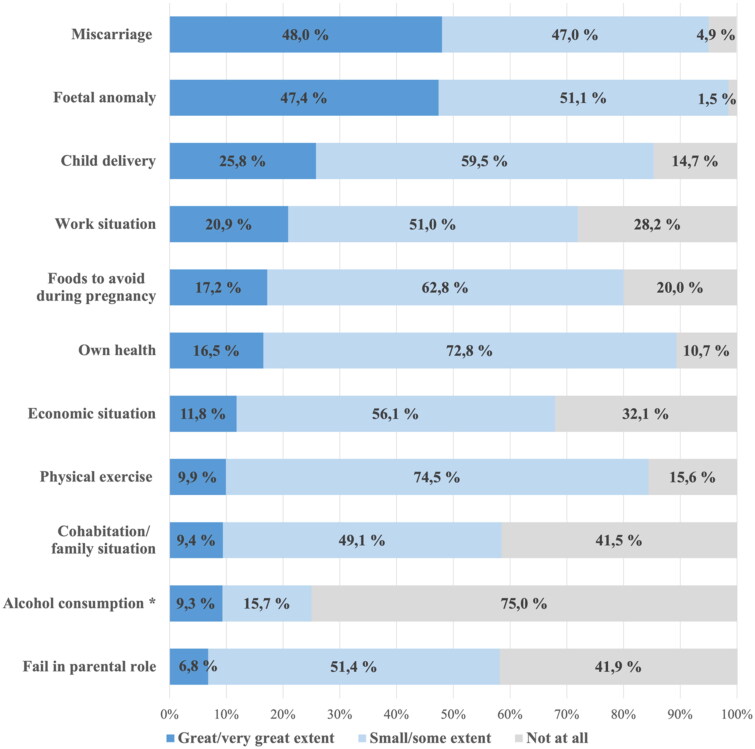
Degrees of worry during the pregnancy so far (*n* = 1399–1402). *Alcohol consumption: worries related to own alcohol consumption before or after the pregnant woman became aware of the pregnancy.

Figure 1 shows the extent of worries regarding 11 specific concerns so far in the current pregnancy. Nearly, all women 99% (*n* = 1381) reported that they were worried about foetal anomalies, and 95% (*n* = 1332) about miscarriage. Furthermore, 85% (*n* = 1195) were worried about delivery and 89% (*n* = 1252) were worried about their own health.

**Figure 2.**
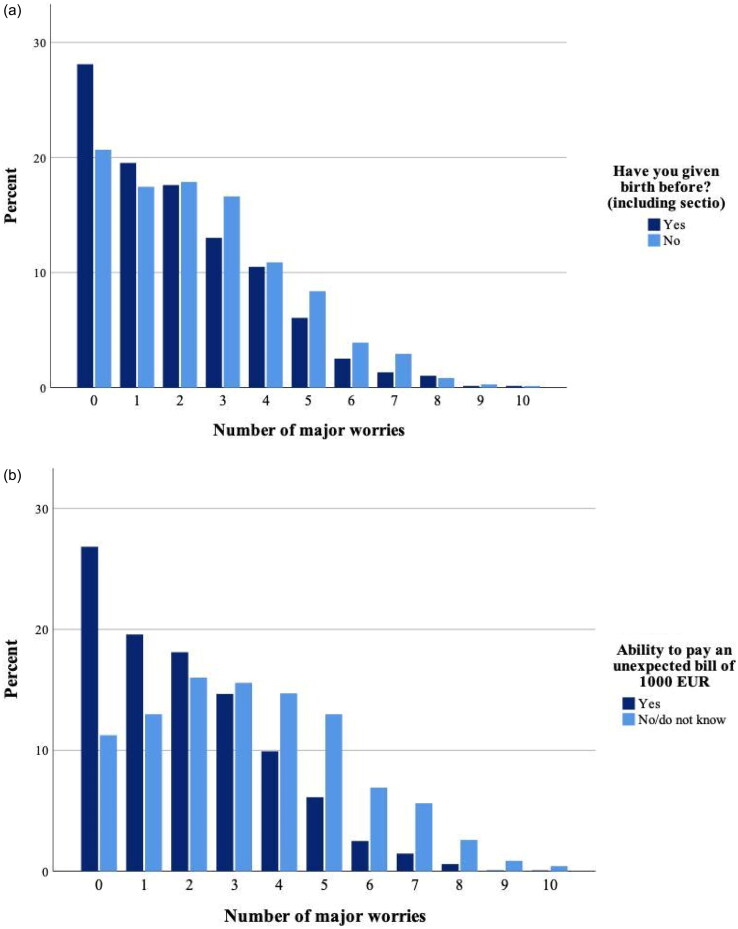
(a) Major worries during pregnancy. Percentage of respondents who reported having experienced worries to a ‘great/very great’ extent from among the list of 11 worries in Figure 1 distributed by parity (*n* = 1392). (b) Major worries during pregnancy. Percentage of respondents who reported having experienced worries to a great/very great from the list of 11 worries in Figure 1, distributed by economic status (*n* = 1390).

Figure 2a,b shows the proportion of the 11 worries each woman reported as a *major* worry (in the sense that they reported worries to a ‘great/very great’ extent, corresponding to the dark (blue/black) colour in Figure 1). In total, 76% (*n* = 1054) had at least one such major worry. This was more common among first-time mothers than among those who had given birth before 79% (568/716) vs. 72% (486/676) (*χ*^2^(1) = 10.5, *p* = .001) (Figure 2a). First-time mothers also reported a greater number of major worries in pregnancy (2.43, 95% CI: 2.28–2.58) compared to women who had given birth before (2.02, 95% CI: 1.87–2.16).

In Figure 2b, the number of major worries in pregnancy so far is distributed by financial security, as defined by the ability to pay an unexpected bill of EUR 1000. Women facing financial insecurity more frequently reported at least one major worry, compared to those with financial security 89% (205/231) vs. 73% (848/1159) (*χ*^2^(1) = 25.4, *p* < .001). Similarly, women facing financial insecurity reported a greater number of major worries: 3.29 (95% CI: 3.00–3.58) vs. 2.02 (95% CI: 1.91–2.13). We also calculated the proportion reporting major worries for each of the 11 specific concerns (see Figure 1 for list) among women with and without financial insecurity, respectively (see Additional file A2). As expected, we found that women facing financial insecurity more frequently reported major worries concerning work and finances. In addition, financially insecure women reported more major worries concerning family situation, miscarriage, foetal anomaly, child delivery and to some extent their own health.

### Worries related to the postpartum period

**Figure 3.**
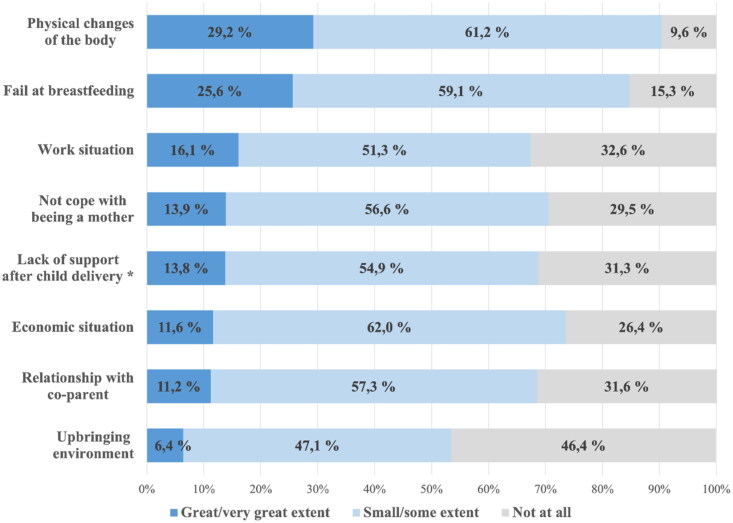
Degree of worry related to conditions after delivery, reported during pregnancy (*n* = 1390–1401). *Lack of support after delivery: not having access to support from others during the first period after delivery.

Figure 3 presents the degree of worry regarding eight specific concerns the pregnant woman had concerning their situation after delivery. The most frequently reported worries were related to potential physical changes to the body (90%, *n* = 1266), breastfeeding (85%, *n* = 1187) and their financial situation (74%, *n* = 1030). Regarding major worries about physical changes to the body, first-time mothers reported greater worries compared to women who had given birth before 34% (244/721) vs. 24% (165/680), as did younger women (<25 years) compared to older women (>37 years) 39% (46/119) vs. 23% (13/57) (see Additional file A3).

**Figure 4.**
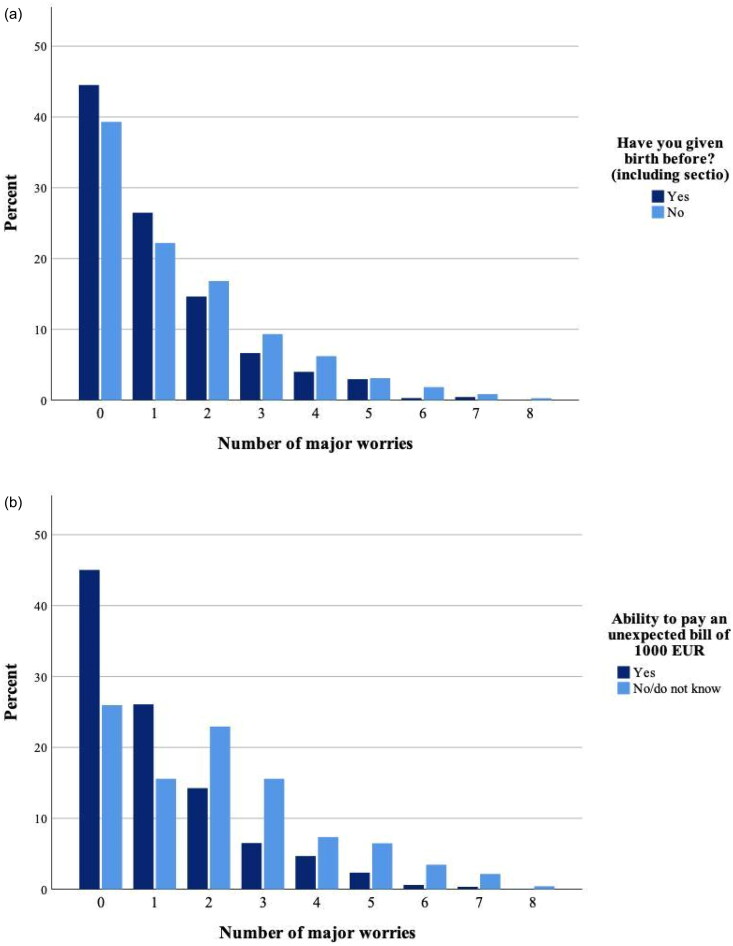
(a) Major worries related to the period after delivery. Percentage of the respondents who reported having experienced worries to a ‘great/very great’ extent from the list of eight worries in Figure 3, distributed by parity (*n* = 1383). (b) Major worries related to the period after delivery. Percentage of the respondents who reported having experienced worries to a ‘great/very great’ extent from the list of eight worries in Figure 3 distributed by economic status (*n* = 1381).

Figure 4a,b shows how many of the eight concerns after childbirth each woman reported as a major worry (in the sense that they were worried to a ‘great/very great’ extent, corresponding to the dark (blue/black) colour in Figure 3). In total, 58% (804/1383) reported having at least one major worry and this was more common among primiparous than multiparous 61% (429/707) vs. 55% (375/676) (*χ*^2^(1) = 3.8, *p* = .05) (Figure 4a). On average, primiparous reported slightly more major worries related to conditions after delivery (1.44, 95% CI: 1.31–1.56) compared to multiparous (1.11, 95% CI: 1.01–1.22).

Figure 4b shows that women facing financial insecurity more frequently reported at least one major worry for the situation after childbirth than those with financial security 74% (171/231) vs. 55% (632/1150) (*χ*^2^(1) = 28.7, *p* < .001). The mean value of reported major worries was again higher for those facing financial insecurity (2.10, 95% CI: 1.85–2.34) than those with financial security (1.11, 95% CI: 1.03–1.20). We also calculated proportions reporting major worries for each of the eight specific items after childbirth (see Figure 3 for list) among women with and without financial insecurity, respectively (see Additional file A4). Women facing financial insecurity had a higher proportion of major worries concerning their economic and work situation, but also concerning the relationship with the co-parent and not coping with being a mother.

### Information during the antenatal consultations

Nearly, all women (99,8%, *n* = 1358) reported attending all or most of the antenatal check-ups so far in the current pregnancy. The majority experienced that the information received during these antenatal consultations was adapted to their needs and interests to a ‘great/very great’ extent 62% (*n* = 872) and 38% (*n* = 529) did not. Very few were dissatisfied with the information given by healthcare personnel (1.2%, *n* = 17). However, one out of five women (20%, *n* = 280) had received information during the antenatal consultations that had generated worries (see Additional file A5).

### Preferred information sources during pregnancy

We investigated how frequently women sought information regarding their pregnancy. Among first-time mothers, 56% (304/721) sought information daily or multiple times per day. In contrast, only 33% (224/678) of women who had previously given birth did the same. Additionally, older women (>37 years old) sought information at least daily slightly more often than younger women (<25 years old): 58% (33 out of 57) compared to 50% (60 out of 119). Information seeking was reported somewhat less frequent during the second trimester (42%, 324/776) compared to the first and third trimesters, which were 45% (45/99) and 50% (258/522), respectively (see Additional file A6).

**Figure 5.**
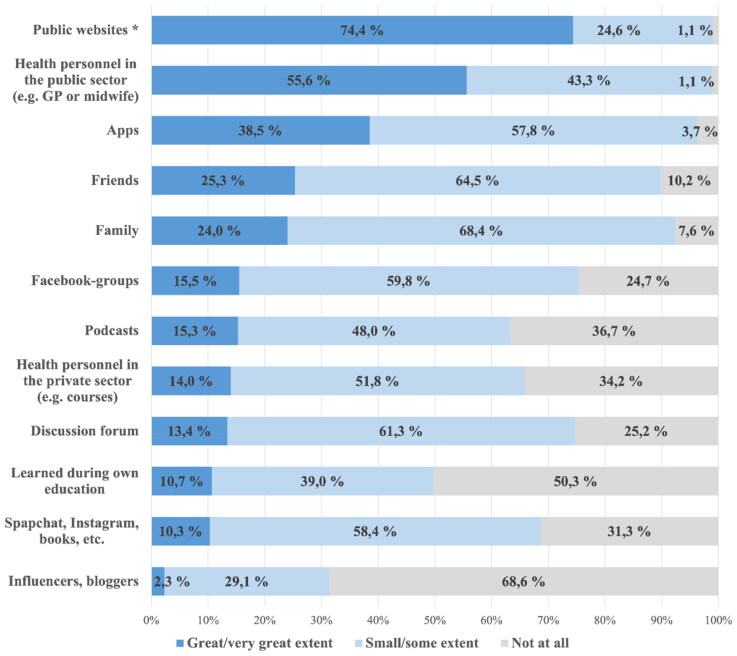
Sources of information used when pregnant women wanted to learn more or had questions regarding the pregnancy (*n* = 1395–1402). *Public websites: quality-assured public healthcare information delivered by government agencies.

Figure 5 shows the information sources used when the women had specific questions or wanted to learn more in general during their pregnancy. Quality-assured public health information websites and healthcare personnel in the public health service (GPs and midwives) were used to a ‘great/very great’ extent by 74% (*n* = 1042) and 56% (*n* = 775), respectively. Only 2% (*n* = 32) sought information from influencers or bloggers to a ‘great/very great’ extent.

However, when in need of personal advice and support regarding pregnancy, 99% (*n* = 1386) turned to healthcare personnel to at least some extent, 92% (*n* = 1288) to family members and 92% (*n* = 1282) to friends. Few approached people online who they did not know personally (see Additional file A7).

## Discussion

### Main findings

This extensive survey of 1402 pregnant women recruited through social media gives an updated overview of pregnancy-related worries and information seeking in Norway. Nearly, all participants expressed worries and concerns related to their pregnancy. The most frequently reported concerns were related to foetal anomalies, miscarriage and childbirth. After delivery, the women were most concerned about physical changes in the body and problems with breastfeeding. First-time mothers and women who experienced financial insecurity tended to be more concerned. Most women relied more on formal quality-assured information channels than on informal sources.

### Methodological strengths and limitations

We consider the face validity of the questionnaire to be good. This is based on a successful pilot test with few remarks, a high participation number and very little missing data in the study. In sum, this suggests that participants found the study relevant and in good accordance with the objective.

The questionnaire was anonymous, and anyone could answer, and even several times. We consider multiple responses from the same participant to be highly unlikely, although this cannot be ruled out. Missing data were negligible and therefore considered to be of little importance to the interpretation of the results.

The study population’s socio-demographic characteristics (age, parity and marital status) appear to be largely comparable with the general population of pregnant women in Norway, except for the proportion of first-time mothers, participants from central Norway and the proportion with higher education, all of whom appeared to be slightly over-represented in our material (see Additional file A8). An important limitation of our study was that only 4.7% of the participants were born outside Norway, compared to 26.6% of women giving birth in Norway, indicating that this group is underrepresented in our study (see Additional file A8).

Beyond sociodemographic representativity, the generalisability of our study findings is difficult to evaluate. The majority (62%) of our respondents were recruited from Facebook groups organised in cohorts according to the estimated month of delivery. Pregnant women who engage actively with such groups over time may potentially have different levels of concerns and seek information in a different manner, compared to women who visit social media platforms more occasionally or hardly at all. Women with greater worries may also have been more likely to participate than those less worried, thereby somewhat overestimating the prevalence of concerns compared to pregnant women in general. While this potential self-selection bias might limit the generalisability of our findings to the broader population of pregnant women, the high number of participants enhances the reliability of our results and provides valuable insights into the concerns and information-seeking behaviours of Norwegian-speaking pregnant women active on social media.

### Findings in relation to comparable studies

Our findings of frequent worries are consistent with earlier research. In line with previous studies, the most frequently reported worries were that something would be wrong with the child, fear of miscarriage and childbirth [6–9,24,25]. However, the proportion worried about miscarriage was higher in our study than reported in a study from 2003 (96% vs. 62%) [7]. Our study also aligns with conclusions from other studies that first-time mothers are more worried than women who have given childbirth before [7,8].

We found anticipatory concerns regarding problematic breastfeeding or difficulties with being a parent to be common, similar to what has been reported by postpartum parents [26–28]. We also found that nine out of 10 participants were worried about physical changes in their bodies after delivery. In the few previous studies in which this issue has been addressed, this was found to be a major concern among pregnant women [25,29]. A recent review by McCarthy et al. [25] found that women worried about self-care during the perinatal period, such as eating correctly and exercising.

Even though Norway has a good welfare system, around 70% of the participants were to some extent concerned about their financial situation before and after childbirth, which was somewhat unexpected. The proportion who expressed such worry is greater than found in studies from Sweden and Germany dating some years back, where approximately half of the pregnant women reported any worry at all about their financial situation [7,8].

Our findings indicate that women who experience financial insecurity tend to have a greater number of major worries than financially secure women, both during their pregnancies and in the period after delivery. The reasons that financially insecure women worry about foetal anomalies, miscarriage, childbirth and family relations are likely to be complex. On the one hand, it is generally easier to tackle major life challenges (including the birth of a disabled child) when your financial circumstances are robust. It is also possible, however, that our findings of increased concerns reflect an awareness of increased risk. A life characterised by a financial struggle represents a risk factor as such and is associated with an increased risk of both pregnancy loss and perinatal death among less educated women [30,31].

The systematic review by McCarthy et al. [25] identified several contributing causes of stress and anxiety in the perinatal period, such as lack of social support, pressure to adhere to social norms and expectations, and poor care from healthcare professionals. The review concludes that better social support and offering customised information are necessary to minimise stress. Our study clearly shows that pregnant women frequently seek information from various sources, and this could indicate that their information needs are not met by healthcare personnel. Although more than half the women in our study reported receiving relevant and adapted information during antenatal care visits, some had received information that generated worries. Promoting the use of reliable public health websites and ensuring that healthcare personnel provide accurate and reassuring information is essential. This might be even more important for women of foreign origin, a group that was poorly represented among the respondents in our study.

In contrast to the recent review by Conrad [17], we found that pregnant women who searched for information mainly turned to formal information channels (e.g. healthcare personnel or quality-assured public healthcare information) and to a lower degree informal information channels (e.g. influencers, bloggers, discussion forums, Snapchat and Instagram). The primary use of quality-assured public websites is reassuring, as pregnant women have previously been reported to consider online resources in general to be trustworthy [16]. Unlike Conrad’s review [17], which grouped search engines with informal information sources, we did not assess the use of search engines. This might partly explain deviating results, as search engines can also guide women to public websites with quality-assured information. Our findings thus nuance the conclusions from Conrad et al. regarding the use of reliable online sources. Their frequent use suggests that public websites are easily identified and contain information considered useful in Norway. Like Sayakhot and Carolan-Olah [16], we found that women seek information more frequently in their first pregnancy than in later pregnancies.

## Conclusions

Worries related to pregnancy and the period after childbirth are widespread and seem to be the norm. First-time mothers and women facing financial insecurity reported a larger number of major worries. Most pregnant women seem to favour information from quality-assured websites and healthcare personnel. Public websites thus seem to be a useful information channel to increase the health awareness of pregnant women.

For healthcare personnel, it is crucial to recognise the normality of worries and offer tailored information and follow-up, particularly for first-time mothers and financially insecure women. Antenatal care could benefit from offering more differentiated care, with a greater focus on first-time mothers. Future research should include a more diverse sample, focusing on pregnant women from different socioeconomic backgrounds and those born outside Norway.

## Supplementary Material

Supplemental Material

## Data Availability

The datasets used and analysed during the current study are available from the corresponding author on reasonable request.
