# Supplementary material for: Worries and information seeking during pregnancy: a cross-sectional study among 1402 expectant Norwegian women active on social media platforms
Source: Scand J Prim Health Care. 2025 Feb 15;43(2):488–99. doi: 10.1080/02813432.2025.2461036 (PMC12090261; doi:10.1080/02813432.2025.2461036)
Supplement: Supplemental Material [file IPRI_A_2461036_SM3612.docx]

**Additional files**

**Additional file A1:** Overview of how we categorized employment status when study participants reported multiple employment statuses simultaneously.

| **Employment statuses simultaneously reported by the respondents** | | | **Categorized as** |
| --- | --- | --- | --- |
| Employed | Student |  | Student |
| Unemployed (job seeker) | Housewife |  | Unemployed |
| On public welfare | Housewife |  | On public welfare |
| Unemployed (job seeker) | On public welfare |  | On public welfare |
| Employed | Housewife |  | Employed |
| Employed | On public welfare |  | On public welfare |
| Employed | Apprentice |  | Student/apprentice |
| Employed | Student | Housewife | Student/apprentice |

**Additional file A2**: Proportions reporting worries in pregnancy to a great/very great extent among women reporting financial security (n=1163-1166) or reporting financial insecurity (n=233-234)

|  | **Financial security** | | **Financial insecurity** | |
| --- | --- | --- | --- | --- |
| **Concerns** | **Proportion** | **95% Cl** | **Proportion** | **95% Cl** |
| Own health | 15.2% | 13.1-17.2 | 23.1% | 17.7-28.5 |
| Foods to avoid during pregnancy | 17.4% | 15.2-19.6 | 16.2% | 11.5-21.0 |
| Alcohol consumption | 9.3% | 7.6-10.9 | 9.4% | 5.7-13.1 |
| Physical exercise | 10.0% | 8.3-11.8 | 9.4% | 5.7-13.2 |
| Cohabitation/ family situation | 7.8% | 6.3-9.4 | 17.5% | 12.3-21.9 |
| Work situation | 17.4% | 14.8-19.1 | 38.5% | 32.2-44.7 |
| Miscarriage | 45.8% | 43.0-48.7 | 59% | 52.7-65.3 |
| Foetal anomaly | 44.5% | 41.7-47.4 | 61.5% | 55.3-67.8 |
| Child delivery | 23.4% | 21.0-25.8 | 38.2% | 32.0-44.4 |
| Fail in parental role | 5.6% | 4.3-6.9 | 12.8% | 8.5-17.1 |
| Economic situation | 5.4% | 4-1-6.7 | 43.8% | 37.4-50.1 |

**Additional file A3**: *Level of worries during pregnancy related to physical changes of the body during childbirth distributed by parity and age of the respondents (n = 1399-1401)*


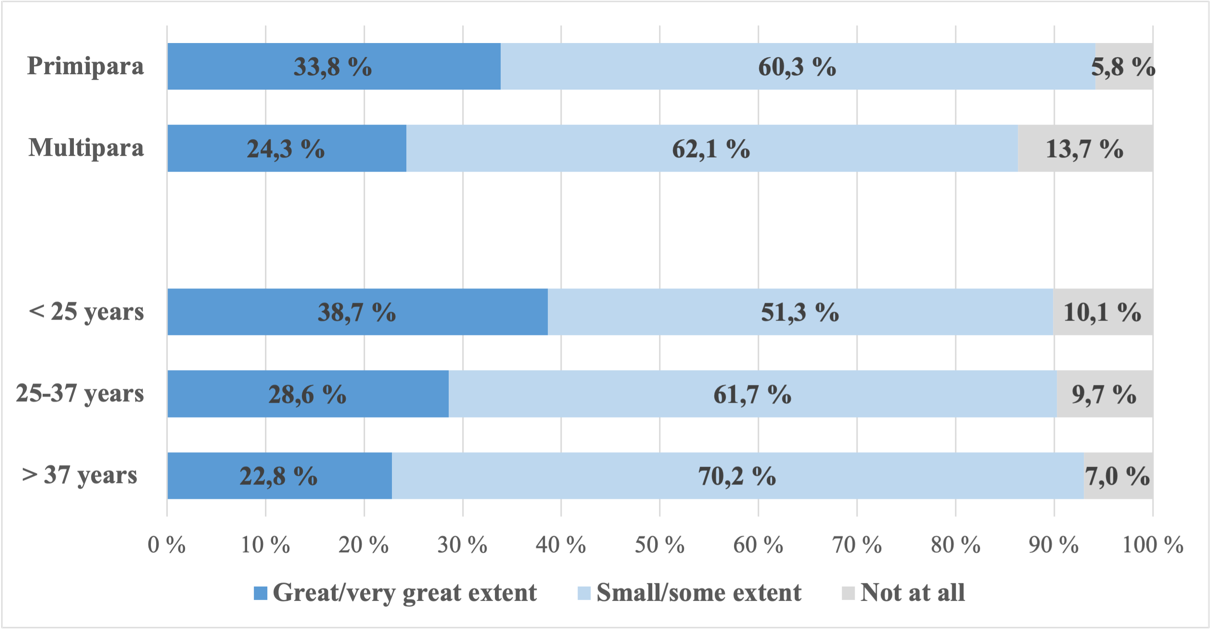


**Additional file A4:** Proportions reporting worries for the situation after delivery to a great/very great extent among women reporting financial security (n=1157-1165) or reporting financial insecurity (n=231-234)

|  | **Financial security** | | **Financial insecurity** | |
| --- | --- | --- | --- | --- |
| **Concerns** | **Proportion** | **95% Cl** | **Proportion** | **95% Cl** |
| Relationship with co-parent | 9.5% | 7.8-11.2 | 19.5% | 14.4-24.6 |
| Economic situation | 5.3% | 4.0-6.6 | 42.7% | 36.4-49.1 |
| Work situation | 11.9% | 10.0-13.7 | 37.6% | 31.4-43.8 |
| Physical changes of the body | 28.7% | 26.1-31.3 | 32.1% | 26.1-38.0 |
| Fail at breastfeeding | 24.6% | 22.2-27.1 | 29.9% | 24.0-35.8 |
| Not cope with being a mother | 12.7% | 10.8-14.6 | 20.1% | 15.0-25.2 |
| Upbringing environment | 5.6% | 4.3-6.9 | 10.7% | 6.7-14.6 |
| Lack of support after child delivery | 13.2% | 11.3-15.2 | 16.2% | 11.5-21.0 |

**Additional file A5**: *Distribution of answers to the questions concerning antenatal care visits*

| Questions |  | n | % |
| --- | --- | --- | --- |
| Have you attended all of your antenatal care visits? | Yes, all  Yes, most of them  No  Not relevant/been to the first control yet | 1323  35  3  40 | 94.4  2.5  0.2  2.9 |
| To what extent was the information you received during your antenatal visits adapted to your needs and interests? | Great/very great extent  Some/small extent  Not at all | 872  512  17 | 62.2  36.5  1.2 |
| Have you been given the opportunity to talk to health personnel about subjects that you find important or former experiences? | Great /very great extent  Some/small extent  Not at all | 885  473  40 | 63.3  33.8  2.9 |
| Do you feel like you have been given sufficient information from health personnel this far during your pregnancy? | Great /very great extent  Some/small extent  Not at all | 845  529  23 | 60.5  37.9  1.6 |
| Have you been given advice or information during an antenatal visit which made you worried? | Yes  No | 280  1117 | 20.0  80.0 |

**Additional Figure A6**: *How often the respondent search for or reads information about pregnancy/childbirth or being a parent distributed by trimester, parity and age of the respondents (n = 1397-1399)*


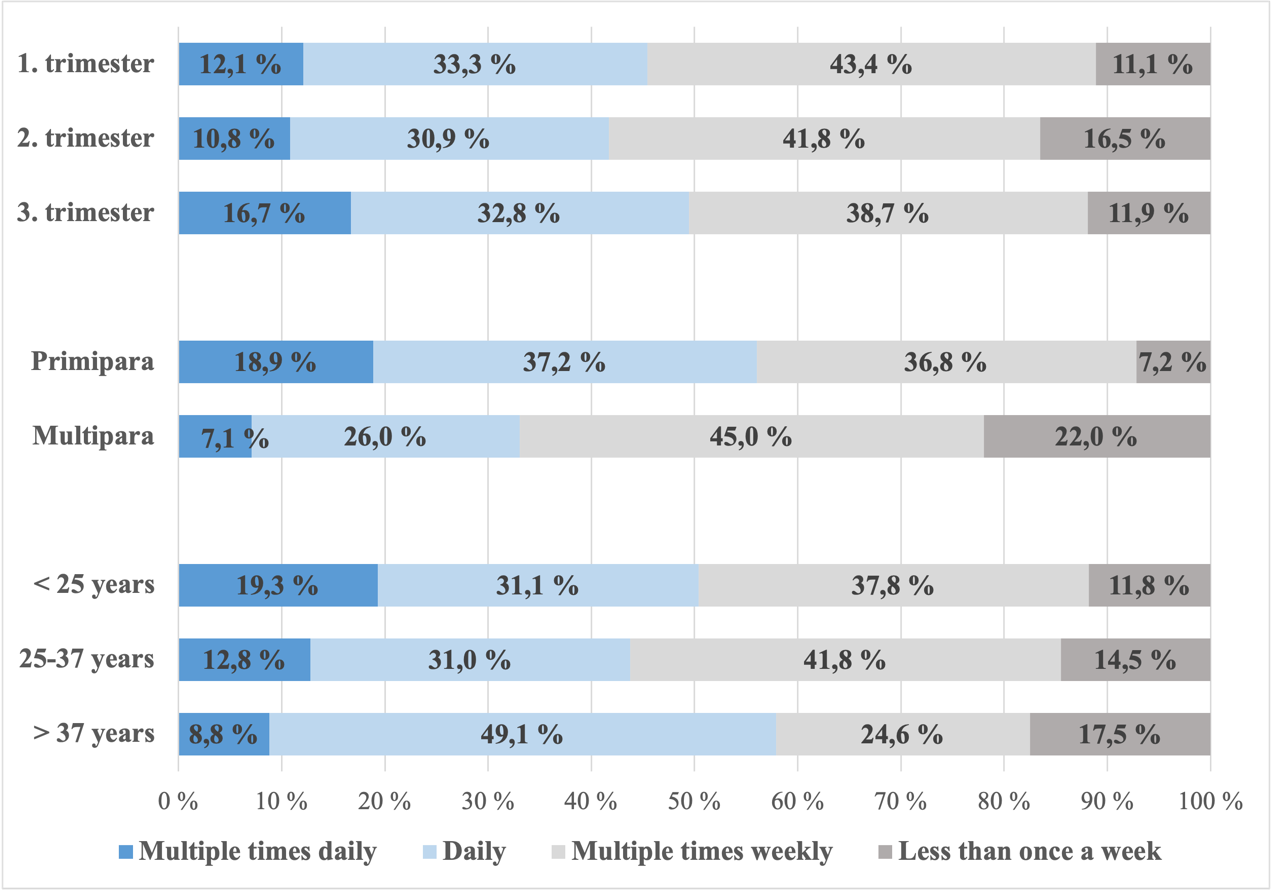


**Additional file Figure A7**: *Who does the respondent ask for advice and support related to the pregnancy (n = 1400-1402. Others: n = 1387). * People online that I do not know personally.*


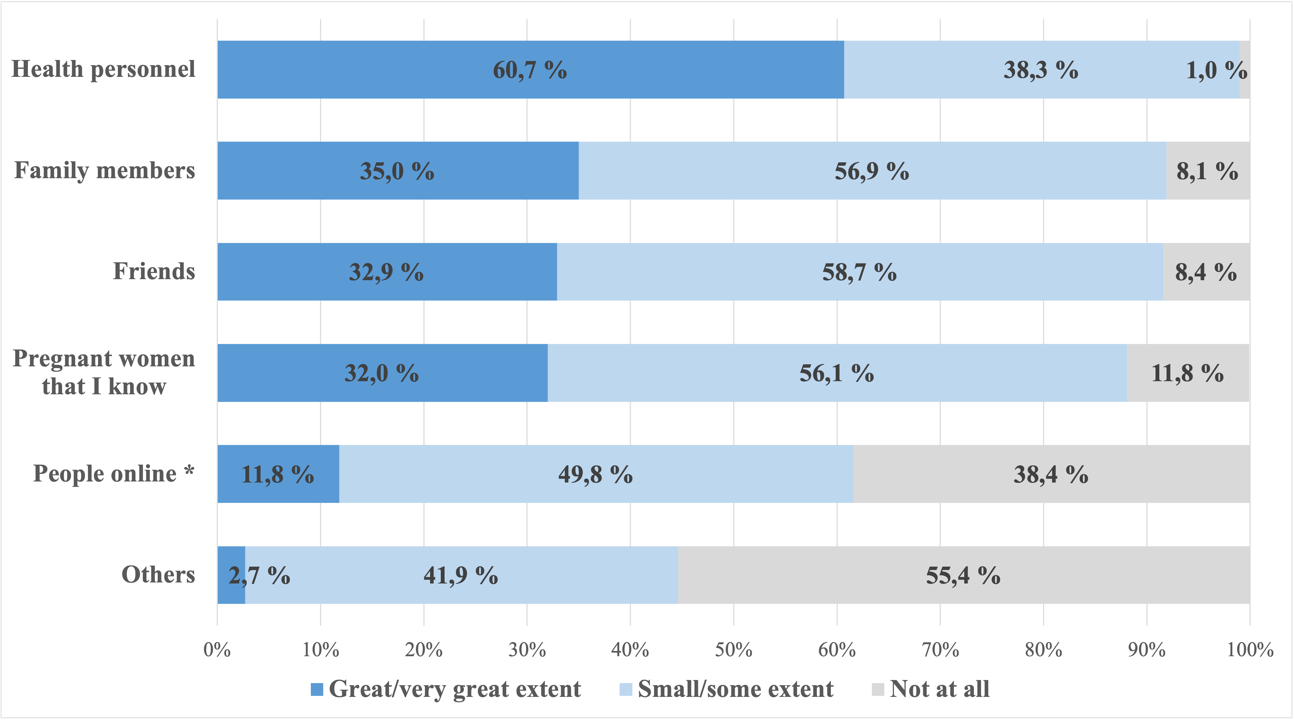


**Additional file Table A8**: *Demographics characteristics of the pregnant women who gave birth in Norway in 2021, based on publicly available data from the Norwegian Medical Birth Registry (*<http://statistikkbank.fhi.no/mfr/>)

| **Variables** |  | **n** | **%** |
| --- | --- | --- | --- |
| **Age**  (n = 55 892) | < 25  25-34  35-39  > 39 | 4 261  39 183  10 238  2 210 | 7.6  70.1  18.3  4.0 |
| **Parity**  (n = 55 892) | Primipara  Multipara | 23 751  32 141 | 42.5  57.5 |
| **Marital status**  (n = 55 892) | Married/cohabiting  Other | 52 762  3 123 | 94.4  5.6 |
| **Residence**  (n = 56 671) | Northern Norway*  Central Norway*  Western Norway*  Eastern Norway*  Southern Norway* | 4 727  7 641  12 450  24 909  6 755 | 8.3  13.5  22.0  44.0  11.9 |
| **Maternal birth place**** | Norway  Other country |  | 73,4  26,6 |

** Northern Norway includes counties Finmark, Troms and Nordland. Central Norway includes counties Trøndelag and Møre og Romsdal, Western Norway includes counties Vestland and Rogaland, Eastern Norway includes counties Viken, Oslo and Innlandet, and Southern Norway includes counties Telemark, Vestfold and Agder.*

***Numbers from; Ottesen HS, Sørbye IK, Lindskog BV, Vangen S, Sundby J, Owe KM. Caesarean sections among immigrant women with different levels of education. Tidsskr Nor Laegeforen. 2022, 21;142(17).*
